# Rule-based systems to automatically count bites from meal videos

**DOI:** 10.3389/fnut.2024.1343868

**Published:** 2024-05-17

**Authors:** Michele Tufano, Marlou P. Lasschuijt, Aneesh Chauhan, Edith J. M. Feskens, Guido Camps

**Affiliations:** ^1^Division of Human Nutrition and Health, Wageningen University & Research, Wageningen, Netherlands; ^2^Wageningen Food and Biobased Research, Wageningen University & Research, Wageningen, Netherlands; ^3^OnePlanet Research Center, Plus Ultra II, Wageningen, Netherlands

**Keywords:** eating behavior, computer vision, video analysis, rule-based system, 3D facial key points

## Abstract

Eating behavior is a key factor for nutritional intake and plays a significant role in the development of eating disorders and obesity. The standard methods to detect eating behavior events (i.e., bites and chews) from video recordings rely on manual annotation, which lacks objective assessment and standardization. Yet, video recordings of eating episodes provide a non-invasive and scalable source for automation. Here, we present a rule-based system to count bites automatically from video recordings with 468 3D facial key points. We tested the performance against manual annotation in 164 videos from 15 participants. The system can count bites with 79% accuracy when annotation is available, and 71.4% when annotation is unavailable. The system showed consistent performance across varying food textures. Eating behavior researchers can use this automated and objective system to replace manual bite count annotation, provided the system’s error is acceptable for the purpose of their study. Utilizing our approach enables real-time bite counting, thereby promoting interventions for healthy eating behaviors. Future studies in this area should explore rule-based systems and machine learning methods with 3D facial key points to extend the automated analysis to other eating events while providing accuracy, interpretability, generalizability, and low computational requirements.

## Introduction

Eating behavior plays a key role in determining nutritional intake of people and is defined by food choices, eating habits and food oral processing (bites, chews, and swallows). Eating behavior is shaped throughout life by a combination of parent–child interactions, peer influences, interaction with the food environment, and the textural properties of food (1–3). An example of individual eating behavior is the eating rate (food in grams/min), which has been shown to impact food intake (4), energy intake (5), and weight gain (6, 7). Indeed, a fast eating rate can increase the risk of obesity (8, 9) and metabolic diseases (10, 11). Therefore, measuring individual eating behavior is needed to support individual interventions to decrease eating rates and the risk of obesity (12, 13).

To measure eating behavior, eating episodes (i.e., a meal) must be analyzed to count eating behavior events. To achieve this, video recordings are an essential and non-invasive source of information. Currently, two trained researchers must watch the videos to annotate every eating event manually and compare their results to ensure an acceptable level of consistency (14). Due to the repetitive and time-consuming nature of this task, manual annotation is prone to subjectivity and attentional lapses (15). The current method does not allow large prospective studies and real-time feedback on individual eating behavior. To improve eating behavior research and perform research in larger cohorts, the human annotation process should be automated with computer technologies, such as face detection (16).

Face detection methods are used to recognize human faces and facial features in an image or video sequences. Key points (or landmark) detection is a computer vision task to localize and track key points on the human face and body from a camera or videos. Several 2D key point detectors and packages are available for face and facial features detection: Viola-Jones face detector (17), Kazemi-Sullivan key point detector (18), OpenSMILE (19), OpenFace (20), OpenPose (21), and dlib (22). A novel 3D face detector, Mediapipe can detect human faces and apply 468 key points to it (compared to the 68 key points of 2D detectors) (23). These open-source packages can be tailored to solve a given task through machine learning or a rule-based system.

Machine learning approaches with facial key points have been used to detect eating behavior events from video recordings of eating episodes. For example, to predict difficulty of speaking while eating, mouth key points can be used to train support vector machines and deep neural networks algorithms for classification (24, 25). To count bites, the mouth corner between upper and lower lips can be calculated through mouth key points, and deep neural networks can be trained for the classification task (26). To detect bites, mouth corners and upper body key points can be identified through key points to develop a deep learning-based algorithm for classification (15).

Facial key points can be coupled with rule-based systems, which employ the conditional “if-then” logic sequences to process inputs and execute decisions within a set of predetermined rules. In rule-based systems, the Euclidean distance is often employed to quantify the separation between two specific reference key points. This measurement serves as the foundation for establishing rules that classify various eating behaviors. For instance, the Euclidean distance between a reference key point on the left eye and another on the jaw region can be calculated to detect chewing (27). Similarly, the distance between key points on hands and mouth can be used to detect eating, when the hands are near the mouth (28).

Facial key points are the optimal method to automatically count bites from video recordings, (29). While existing techniques primarily rely on 68 2D facial key points for this purpose, the advantages of utilizing 3D facial key points have not yet been explored for bite detection. Therefore, this study aimed to assess the accuracy of a system that uses 468 3D facial key points in detecting bites from video recordings. Our focus was on identifying the least computationally expensive and most versatile solution to implement, which led us to explore key-point-based decision models, and to assess their viability and effectiveness in detecting bites. To our knowledge this study is the first to apply rule-based system with 3D facial key points to a large video dataset.

## Methods

### Study participants

The study was performed at Wageningen University and Research (the Netherlands), Human Nutrition Research Unit, between October and December 2020. Master thesis students and personnel of the Division of Human Nutrition and Health were not allowed to participate in the study. Healthy adults (18–55 years, BMI: 18.5–30 kg/m2) were recruited as participants through the divisional volunteer database and advertisements on social media. The participants signed informed consent and filled in the inclusion/exclusion questionnaire. The inclusion criteria were English proficiency, and normally eating three meals per day during weekdays. In contrast, the exclusion criteria were food allergies to the foods used in this study, a lack of appetite, chewing or swallowing problems, following an energy-restricted diet, more than 5 kg weight change during the last 2 months, alcohol consumption higher than 21 glasses per week, being on medications influencing appetite, taste or smell, intensive exercise for more than 8 h a week, and being a high restrained eater (according to the Dutch Eating Behavior Questionnaire (30)). In total, 58 participants were eligible to participate in the study, of which 18 participants (11 females) were selected according to the abovementioned criteria and included in the study. The Social Ethical Committee of Wageningen University (the Netherlands) approved this study (Lasschuijt, 2020–11). The participants received financial compensation.

### Meals and conditions

The participants ate four breakfasts, lunch, dinner, and desserts in the diner/dining room of the behavior research unit for 4 days. The participants were instructed to eat as much or as little as they wanted until they felt comfortably full. For the breakfast meals, the participants were provided with one of the four distinct options: (1) fresh mixed fruit, (2) homemade smoothie, (3) canned mixed fruit, and (4) store-bought smoothie. For the lunch meals, the participants were provided with one of the four distinct options: (1) fresh tagliatelle pasta with homemade tomato sauce, hard-steamed vegetables, and large pieces of chicken fillet, (2) fresh tagliatelle pasta with homemade tomato sauce, soft-steamed vegetables, and homemade pulled chicken, (3) store-bought pork meat tortellini with pre-canned tomato sauce, hard-cooked vegetables, and grated cheese, and (4) ready-to-eat macaroni Bolognese with grated cheese. For the dinner meals, the participants were provided with one of the four distinct options: (1) potato parts with large pieces of pork fillet and whole hard-steamed green beans, (2) homemade mashed potato with eggs small pieces of soft-steamed green beans, (3) pre-flavored and baked potato parts with large pieces of chicken schnitzel and whole, hard-steamed green beans, (4) mashed potato with chicken meatballs and small pieces of soft-steamed green beans. For the dessert meals, the participants were provided with one of the four distinct options: (1) dried figs and almonds, (2) curd with added honey and crushed pecan nuts, (3) mass produced fig bread, (4) walnut and honey flavored yoghurt. According to the NOVA classification (31), two food conditions were chosen: unprocessed (category 1), processed/ultra-processed (category 3 and 4). Food texture was manipulated to create two more conditions: slow and fast. The slow condition included foods that would require small bites, many chews and therefore long oro-sensory exposure duration. Conversely, the fast condition included foods that would require large bites, fewer chews and therefore shorten the oro-sensory exposure duration. Food texture manipulation included solid/liquid manipulation (i.e., fresh fruit vs. smoothie), or hardness and piece size manipulation (i.e., potato parts vs. homemade mashed potato).

### Video recordings

While eating a meal, the participants were recorded with a video camera (Axis M1054, Axis Communications) in front of their seat. The camera was positioned at approximately 1.5 m from the participant, with the lower frame in line with the table, the upper frame above the top of the cranium, and the sides of the frame at shoulder width. The videos were recorded using the software Noldus Observer XT 11 (Noldus Information Technology, the Netherlands) on Windows 10, installed on laptops (Lenovo Thinkpad L380, Intel core i5). To support the video analysis, the participants were instructed to show a numbered card to the camera before they started to eat and to raise a hand once they finished eating. The video recordings were later used for manual annotation and video analysis.

### Manual annotation

The video recordings were annotated by using the software Noldus Observer XT 11 (Noldus Information Technology, the Netherlands). Two human annotators watched the videos and annotated the following eating behaviors: meal duration (min), duration between the bites (s), oro-sensory exposure (s), number of chews, and number of bites during the meal. The annotators calculated the bite size by dividing the total amount of food eaten (in grams) by the number of bites per meal. The eating rate (g/min) was then calculated by dividing the amount eaten (g) by the meal duration (min). The videos recordings from 3 participants could not be analyzed due to technical errors. In total, 170 videos from 15 participants were annotated.

### Video analysis

The video recordings files were opened in Microsoft Photos on Windows 10 (Microsoft, Reedmond, WA, United States), and the video clip editor function was used to cut the video recordings. The videos were cut precisely to capture the entire eating process, beginning the moment the participant showed a numbered card before and ending when they set the ear-sensor on the table. While the ear-sensor was used to track jaw movement, data from this device was not included in the present study. The video recordings from the liquid breakfast meals (homemade smoothie and store-bought smoothie) were discarded for all participants because they were liquid therefore did not include bites.

The software implementation was achieved using Windows 10 on a Lenovo laptop (00329–00000-00003-AA-1666) with an Intel® Core™ i5-8265U CPU @ 1.60 GHz 1.80 GHz, 8.00 GB RAM. The programming language used for video analysis was Python 3.9 (32), with PyCharm 2021.2.2 (Community Edition, version 11.0.12 + 7-b1504.28, JetBrains) as IDE (33). We used Mediapipe for 3D facial recognition (23) using the OpenCV computer vision library (34), and CVZone for visualization (35). NumPy (36) was used to convert a list of facial coordinates into an array and Pandas (37) to store the outcome in a dataset.

### Threshold search

We extracted mouth coordinates from the video using Mediapipe with 3D key points. The mouth ratio is calculated by dividing the Euclidean distance between the upper and lower lips by the distance between the left and right sides of the mouth (key points 0, 17, 61, and 291, respectively). The mouth ratio was extracted from every frame of the meal video recordings. After performing outlier removal based on Z-Scores per participant, we discarded the meals with less than 10 bites (e.g., desserts). We used grid search, random search, and Bayesian optimization to find a custom threshold per participant.

We designed a custom model encapsulated in a Python class, to predict bite counts based on a predefined threshold value. The model does not require training and uses the threshold to count the bites in each sample that exceeds this value. Hyperparameter tuning is performed using Grid Search, Randomized Search, and Bayesian Optimization Cross-Validation methods to optimize the threshold parameter for each unique participant in our dataset. We used cross-validation folds set between 2 and 5 based on the number of samples. The performance was evaluated using a custom scoring function that calculates the negative mean of the absolute differences between the predicted and manually annotated bite counts. The best threshold values obtained for each participant were recorded and stored in a dataset to calculate accuracy in counting bites (Supplementary Table S1).

### Dataset

The final dataset consisted of the total bites count from the manual annotation and bites predictions over videos in 15 participants for 6,719 annotated bites. Descriptive statistics per participant, eating condition, and manual annotation can be found in Tables 1–3, respectively.

**Table 1:** Number of videos, average bite, and standard deviation per participant.

Participant	Videos	Avg bites	Std bites
A	10	55.4	17.3
B	14	35.9	15.4
C	14	39.4	20.1
D	13	34.6	24.9
E	11	59.7	28.2
F	6	40.2	21.0
G	14	34.1	21.7
H	14	43.4	22.6
I	13	34.1	16.0
L	13	51.4	28.2
M	7	49.9	25.7
N	12	32.4	16.2
O	8	22.4	12.1
P	9	45.1	16.0
Q	6	40.7	11.5

**Table 2:** Number of videos, average bite, and standard deviation per eating episode.

Eating episode	Videos	Avg bites	Std bites
Breakfast	25	43,4	18,49
Dessert	38	43,13	26,4
Dinner	50	37,82	20,36
Lunch	51	41,25	21,62

**Table 3:** Descriptive statistics of the manual annotation of bites.

Mean	40.97
Median	40
Mode	50
Range	95
Variance	481.7
Standard deviation	21.95
25th Percentile (Q1)	23
50th Percentile (Q2)	40
75th Percentile (Q3)	56

### Counting bites

We utilized a Python-based video processing pipeline to automatically count bites during eating events. The pipeline employs the CVZone library’s “FaceMeshDetector” for facial landmark detection in each video frame (35). Mouth key points are then extracted and used to calculate the mouth ratio. This ratio is stored and averaged over a window of 5 frames to smooth out noise. Bites are counted based on a predefined threshold for this mouth ratio, found as described above. To prevent double-counting, consecutive frames exceeding the threshold are considered as a single bite event, managed by an internal counter within our custom class. Each bite event is timestamped and recorded for further analysis. The system could process multiple videos concurrently through the multiprocessing module. The accuracy was determined by comparing the number of bites predicted per video with the manual annotations, as outlined in (38). The accuracy of total bite prediction is calculated on the overall number of bites per meal, rather than on the identification of each bite. To estimate the thresholds for participants lacking annotations, we used leave-one-out cross validation (LOOCV). We computed the mean threshold using values obtained through random selection from all other participants, excluding the one under evaluation. In each LOOCV iteration, the threshold was calculated by averaging the remaining 14 threshold values from the random search, omitting one value for validation. This yielded 15 different average thresholds, each leaving out a unique value.

## Results

**Figure 1:**
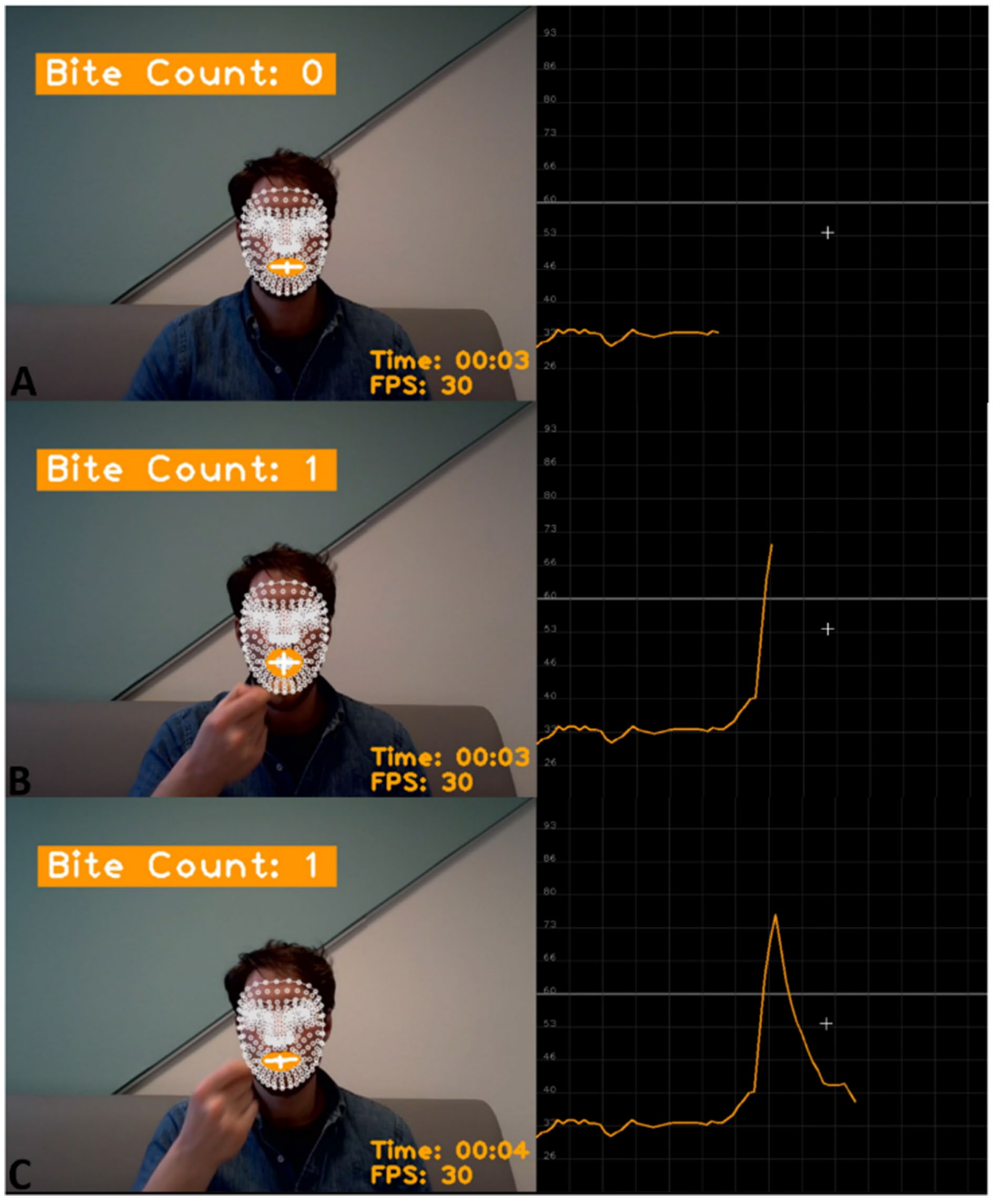
Example of the program counting a bite. On the left: the program displays the current bite count, the time from the start of the video, and the frames per second (FPS). The text is displayed on the video of a participant eating a meal with the facial key points applied to the face (in white), the lips highlighted (in orange), and the vertical and horizontal mouth lines that are used to calculate the mouth ratio (in white); On the right: the horizontal grey line represents the threshold for counting bites. The orange line represents the mouth ratio. **(A)** The mouth ratio is below the threshold. **(B)** The participant takes a bite by inserting food in the mouth. During mouth opening, the mouth ratio increases and surpasses the threshold; the program counts one bite, and the bite count is updated to one. **(C)** The participant closes the mouth after the bite therefore the mouth ratio is again below the threshold.

This study used thresholds for facial key points to count bites from video recordings. We found thresholds per participant using grid search, random search, and Bayesian optimization and applied them to 164 video recordings of eating episodes from 15 study participants. The system counted a bite when the mouth ratio (the distance between the upper and lower lips to the width of the mouth) exceeded the threshold (Figure 1). The videos were manually annotated to provide the ground truth for the bite counts. We evaluated the accuracy of the three methods and determined the system’s accuracy in a scenario where video annotations are unavailable. Further, we tested the system’s accuracy on different meal textures.

Overall, the threshold obtained with the random search achieved the highest accuracy of 79.8% (std: 15.2, min: 47.5%, max: 98.4%). The thresholds obtained with the grid search achieved 76.4% accuracy (std: 18.6, min: 33.9%, max: 99.2%), while the Bayesian optimization approach achieved 72.9% accuracy (std: 18.4, min: 28.4%, max: 99.6%) (Table 4).

**Table 4:** Bite count and accuracy per participant and method, including manual annotation.

		Grid search		Random search		Bayesian optimization	
Participant	Annotation	Predicted bites	Accuracy	Predicted bites	Accuracy	Predicted bites	Accuracy
A	554	524	94.6	532	96.0	369	66.6
B	502	662	68.1	647	71.1	1,334	65.7
C	552	917	33.9	597	91.8	947	28.4
D	450	521	84.2	516	85.3	519	84.7
E	657	490	74.6	572	87.1	490	74.6
F	241	214	88.8	193	80.1	618	56.4
G	478	520	91.2	516	92.1	525	90.2
H	607	594	97.9	559	92.1	620	97.9
I	443	327	73.8	328	74.0	327	73.8
L	668	1,031	45.7	1,019	47.5	958	56.6
M	349	254	72.8	220	63.0	252	72.2
N	389	354	91.0	353	90.7	412	94.1
O	179	256	57.0	264	52.5	256	57.0
P	406	513	73.6	504	75.9	507	75.1
Q	244	246	99.2	240	98.4	243	99.6
Total	6,719	7,423		7,060		8,377	
Average	447.9	494.9	76.4	470.7	79.8	558.5	72.9
Standard deviation			18.6		15.2		18.4

**Figure 2:**
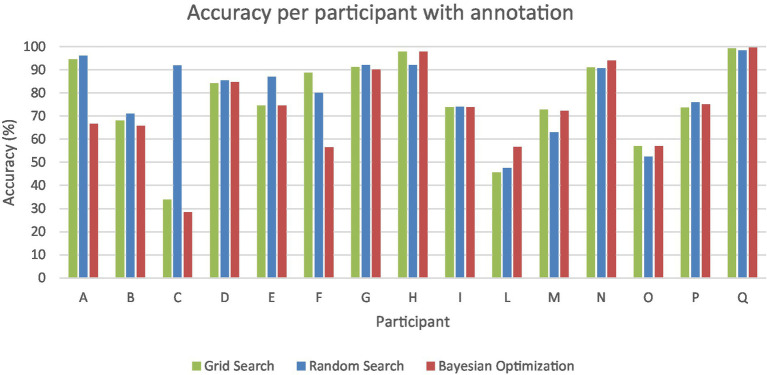
Accuracy in percentage (*y*-axis) per participant (*x*-axis) with annotation for grid search (green), random search (blue), and Bayesian optimization (red).

Figure 2 illustrates the range of performance outcomes across participants for the three methods under consideration. The system showed the highest performance in one participant, with an average accuracy of 99% across the three methods. This was followed by 3 participants, who achieved accuracies of 96, 92, and 91%, respectively. On the other hand, some participants experienced subpar performance. Three out of 15 participants displayed below-average accuracy of 55.8, 51.4 and 49.9%. Notably, one participant exhibited significant variability in the performance of the three methods: grid search yielded an accuracy of 33.9%, random search achieved 91.8%, and Bayesian optimization resulted in 28.4%.

**Figure 3:**
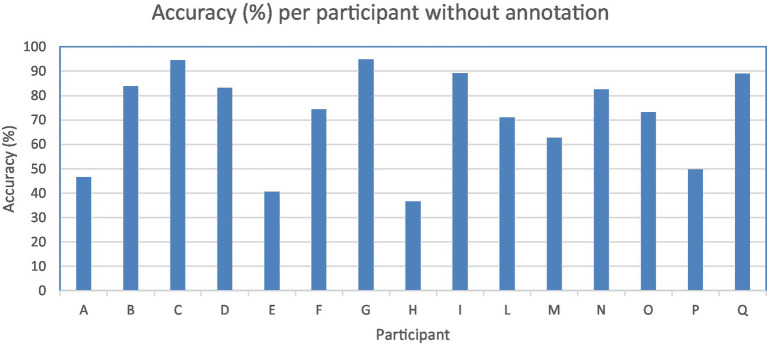
Accuracy in percentage (*y*-axis) per participant (*x*-axis) without annotation.

To assess accuracy when video annotation is unavailable for a participant, we employed leave-one-out cross-validation. The overall accuracy per participant without annotation is 71.4% (std: 19.1, min: 36.6%, max: 94.6%). Half of the participants showed accuracy higher than 80%. The system performed poorly in three participants out of 15 with 46.6, 40.5, and 36.6% accuracy (Table 5; Figure 3).

**Table 5:** Accuracy per participant without annotation, leave-one-out cross validation.

Participant	Accuracy
A	46,6
B	83.9
C	94.6
D	83.1
E	40.5
F	74.3
G	94.8
H	36.6
I	89.2
L	71.0
M	62.8
N	82.5
O	73.2
P	49.8
Q	88.9
Average	71.4
Standard deviation	19.1

**Figure 4:**
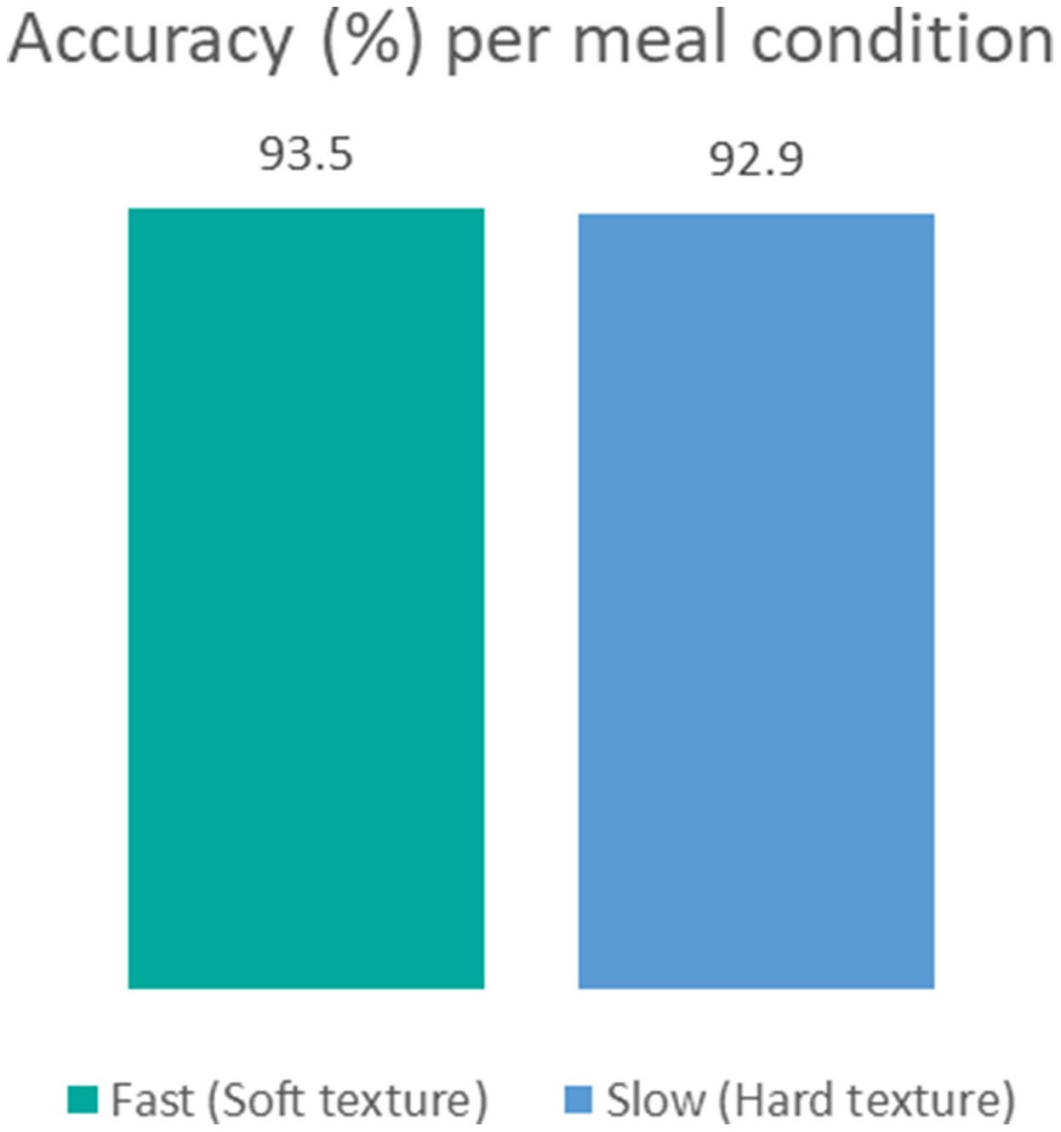
Accuracy in percentage per meal texture condition in fast condition with soft texture (green) and slow condition with hard texture (blue).

**Figure 5:**
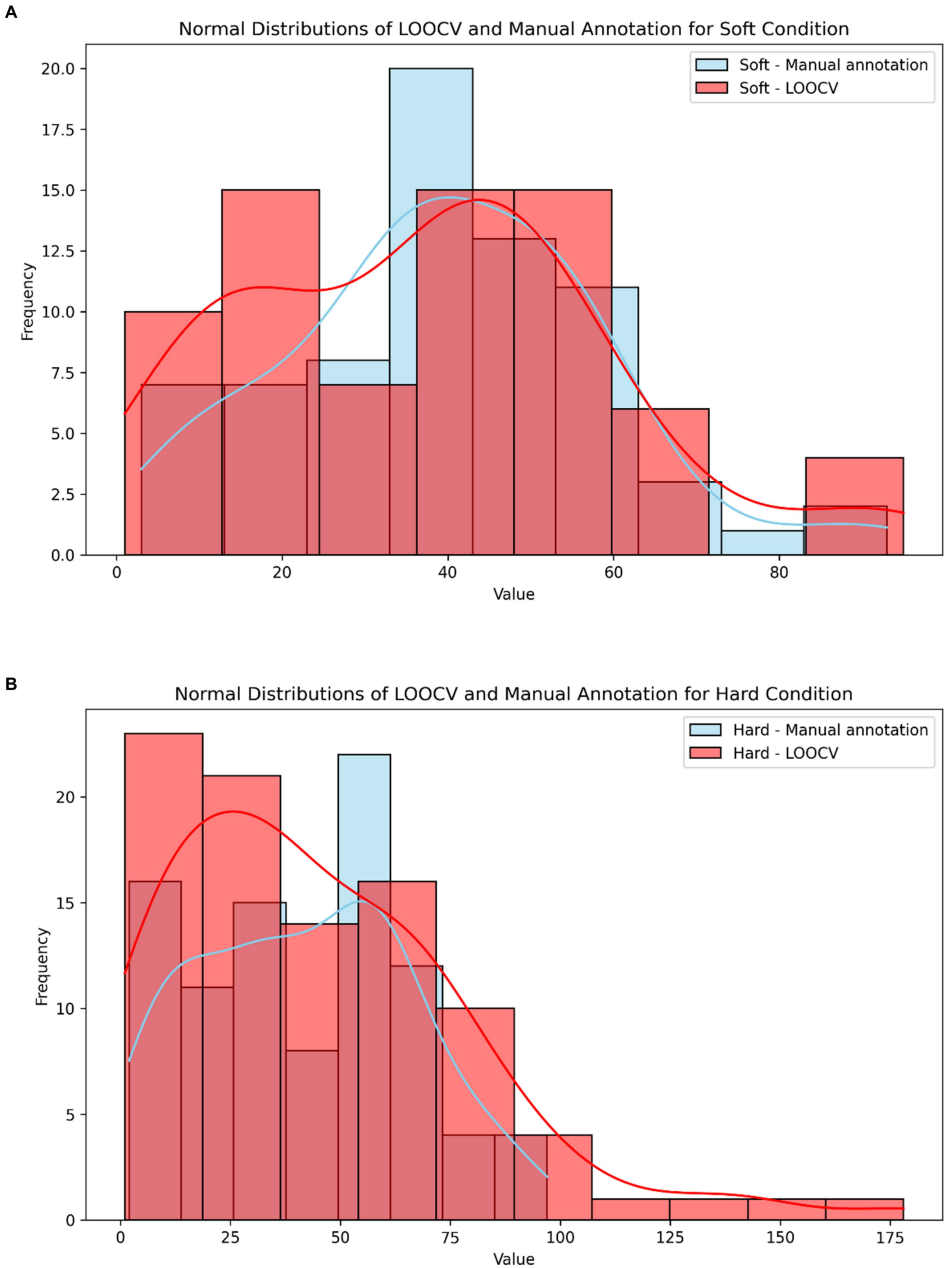
**(A)** Normal distribution of LOOCV (light red) and manual annotation (light blue) for the soft texture. **(B)** Normal distribution of LOOCV (light red) and manual annotation (light blue) for the hard texture.

To assess accuracy when food texture conditions are different, we calculated the system’s accuracy for meals with soft or hard textures. The results revealed negligible differences: 93.5% accuracy for soft meals and 92.9% for hard meals (Table 6; Figure 4). Furthermore, Spearman’s rank correlation coefficient for LOOCV versus manual annotation revealed a slightly stronger correlation in the hard texture than in the soft texture (ρ = 0.7518 and *ρ* = 0.7102), respectively, Figure 5.

**Table 6:** Bite counts and accuracy per food texture condition.

Condition	Manual annotation	Predicted bites	Accuracy
Fast (Soft texture)	2,871	2,684	93.5
Slow (Hard texture)	3,848	4,120	92.9

## Discussion

This study aimed to equip eating behavior researchers with an automatic, fast, standard, and objective method to automatically count bites from video recordings of eating episodes, using 3D facial key points. Our results demonstrate that bites can be detected without the use of deep learning methods. We tested three methods to find the threshold to count bites per participant: grid search, random search, and Bayesian optimization. With available annotation, the thresholds found with random search achieved the highest accuracy of 79.8%, followed by grid search (76.4%), and Bayesian optimization (72.9%). When annotated bites per participant are unavailable, the system achieved 71.4% accuracy. The system performed with comparable accuracy when the participants consumed a meal with soft or hard texture, thus across a slow and fast eating rate.

The system achieves improved accuracy with the availability of bite annotations in videos. In an eating behavior study, if the researchers count the bites per meal video of a participant, the system can achieve 99% accuracy. For one participant, six meal video recordings were annotated for total bite count, leading to \99% accuracy. This level of precision suggests that for some individuals the system could serve as a reliable substitute for manual annotation methods, offering rapid and standardized measurements of bite counts. Another strength of this study is the system’s consistent performance across hard and soft food textures. In the domain of eating behavior research, it is common to examine how participants respond to foods with different textural properties. The system we developed was not affected by variations in food texture and thus eating rate, thereby functioning as an unbiased tool for counting bites within the context of eating behavior studies. Yet, the system seems to slightly overpredict videos with hard texture food. The accuracy analysis and Spearman’s correlation coefficient showed negligible differences in the system’s accuracy when adapted to different food textures.

There are limitations of this study that should be considered. Firstly, the system showed inconsistent accuracy across participants. For three participants out of 15, a below-average accuracy of 55.8, 51.4 and 49.9% was achieved. Such limitations render the system an unreliable substitute to replace manual annotation in those cases. Without annotated bites, the system’s accuracy plunges from 79.8 to 71.4%. This could be a limitation, when precise bite counts are essential.

The performance variability among participants can be attributed to several factors. The participants for which the system achieved high accuracy (91.17, 95.93, and 91.93%) have a relatively high number of videos, which might provide a stable and generalized dataset to find the threshold. Furthermore, these participants have moderate bites standard deviation (21.7, 22.6, and 16.2, Table 1) suggesting that their eating behavior is not too erratic, which might decrease false bite predictions. Three participants out of 15 showed less accurate results (51.37, 49.93, and 55.5). Two of those participants had a high standard deviation, which suggests that the eating behavior is more variable and hence, more difficult to predict.

Moreover, one participant has an average bite count that is significantly lower than the average of all other participants (22.4 vs. 42.6), which could affect the system’s ability to effectively find the threshold for this case. Overall, the system’s accuracy seems to be less effective (55.5%) for participants with less than 177 annotated bites. However, we did not find a correlation between accuracy and video duration or number of videos (Table S3), which prevents setting a minimum video duration requirement for our method. Lastly, it is possible that some external factors affect the performance of the system. For example, the lightning condition, angle of the camera, and presence of other people in the video frame might affect the system’s ability to count bites. We recommend using this method after advising participants to avoid covering their mouth when eating and looking away from the camera.Future studies should explore several camera angles and lighting conditions.

Until now, researchers have used rule-based systems to detect chewing or eating activity, by calculating distances between facial key points or body parts (27, 28). The previous research employed 68 2D facial key points. Here, we used 468 3D facial key points to count bites per meal. We tested the system in 164 meal videos from 15 participants. To our knowledge, this is the first study to examine the performance of 468 3D facial key points and a rule-based system to automatically count bites from meal video recordings. An advantage of our study was the opportunity to investigate such a system on a large video dataset of 164 videos from 15 participants. Compared to deep learning methods to count bites (38), our system offers greater adaptability and versatility for practical implementations.

Our study raises a number of opportunities for future research.

For instance, 3D facial key points could be used in combination with threshold search to count other eating events, and apply thresholds search to facial features different from the mouth ratio. Furthermore, 3D facial key points could be used in combination with machine learning and deep learning models to count bites and other eating behavior events. For instance, deep learning can predict chewing, an unfeasible task with our approach given the uniform mouth ratios associated with chewing behavior However, deep learning systems have high computational requirements, low interpretability, limited generalizability, and large datasets requirements. These limitations could make deep neural networks less accessible for researchers in eating behavior studies and limit their applicability across different research scenarios.

## Conclusion

Our study shows that rule-based systems that use 468 3D facial key points can be used to count bites from video recordings. The system can count bites with 79% accuracy when annotation is available for a small video subset (i.e., six videos), and 71.4% if it is applied to a new participant when annotation is unavailable. The system showed consistent performance across varying food with soft and hard textures. Compared to deep learning approaches, rule-based methods demand lower computational requirements, offer higher interpretability and generalizability, and require less data. For eating behavior researchers, rule-based methods can serve as an accessible tool that can be applied across different research contexts. Eating behavior researchers can use this tool to replace manual annotation of bite count if the accuracy of 79% with available annotation and 71.4% with unavailable annotation is acceptable for the purpose of their study.

## Data availability statement

The raw data supporting the conclusions of this article will be made available by the authors, without undue reservation.

## Ethics statement

The studies involving humans were approved by the Social Ethical Committee of Wageningen University (the Netherlands) approved this study (Lasschuijt, 2020-11). The studies were conducted in accordance with the local legislation and institutional requirements. The participants provided their written informed consent to participate in this study.

## Author contributions

MT: Conceptualization, Data curation, Formal analysis, Investigation, Methodology, Software, Validation, Visualization, Writing – original draft, Writing – review & editing. ML: Conceptualization, Data curation, Project administration, Resources, Supervision, Visualization, Writing – review & editing. AC: Conceptualization, Supervision, Writing – review & editing. EF: Project administration, Resources, Supervision, Writing – review & editing. GC: Conceptualization, Funding acquisition, Project administration, Resources, Supervision, Writing – review & editing.
