# Supplementary material for: Rule-based systems to automatically count bites from meal videos
Source: Front Nutr. 2024 May 17;11:1343868. doi: 10.3389/fnut.2024.1343868 (PMC11141395; doi:10.3389/fnut.2024.1343868)
Supplement: Supplementary file 1 [file Table_1.docx]

**Supplementary Material**

| Participant | Random Search​ | LOOCV |
| --- | --- | --- |
| A | 75.4 | 61.6 |
| B | 56.1 | 63.0 |
| C | 61.7 | 62.6 |
| D | 63.4 | 62.4 |
| E | 50.6 | 63.4 |
| F | 75.4 | 61.6 |
| G | 61.1 | 62.6 |
| H | 72.9 | 61.8 |
| I | 65.6 | 62.3 |
| L | 52.9 | 63.2 |
| M | 61.7 | 62.6 |
| N | 69.4 | 62.0 |
| O | 62.2 | 62.5 |
| P | 49.7 | 63.4 |
| Q | 59.5 | 62.7 |

Table S1 – Threshold used for counting bites found with random search and LOOCV methods.
