# Supplementary material for: Rule-based systems to automatically count bites from meal videos
Source: Front Nutr. 2024 May 17;11:1343868. doi: 10.3389/fnut.2024.1343868 (PMC11141395; doi:10.3389/fnut.2024.1343868)
Supplement: Supplementary file 2 [file Table_2.docx]

| Participant | Food texture | Eating Episode | Manual annotated bites | Duration (mm:ss) |
| --- | --- | --- | --- | --- |
| A | soft | dessert | 50 | 11:15 |
| A | soft | dinner | 54 | 15:52 |
| A | soft | lunch | 66 | 26:55 |
| A | hard | breakfast | 61 | 21:20 |
| A | hard | dessert | 13 | 08:50 |
| A | hard | dinner | 78 | 27:27 |
| A | hard | lunch | 65 | 31:15 |
| A | soft | dinner | 60 | 24:21 |
| A | soft | lunch | 60 | 24:35 |
| B | soft | dinner | 40 | 14:20 |
| B | hard | dinner | 52 | 23:21 |
| B | soft | dinner | 56 | 16:16 |
| B | soft | lunch | 54 | 18:29 |
| C | soft | dinner | 42 | 10:14 |
| C | soft | lunch | 38 | 14:29 |
| C | hard | dinner | 73 | 10:31 |
| C | hard | lunch | 52 | 20:57 |
| C | soft | dinner | 41 | 16:39 |
| C | soft | lunch | 58 | 16:22 |
| C | hard | breakfast | 36 | 09:57 |
| C | hard | dinner | 59 | 21:59 |
| C | hard | lunch | 63 | 16:48 |
| D | soft | dinner | 50 | 13:33 |
| D | soft | lunch | 55 | 13:10 |
| D | hard | breakfast | 33 | 13:04 |
| D | hard | dessert | 10 | 07:14 |
| D | hard | dinner | 83 | 20:30 |
| D | hard | lunch | 57 | 23:32 |
| D | soft | dinner | 37 | 09:30 |
| D | soft | lunch | 33 | 13:14 |
| D | hard | breakfast | 12 | 07:06 |
| D | hard | dessert | 11 | 07:57 |
| D | hard | dinner | 58 | 18:55 |
| E | soft | dessert | 35 | 05:23 |
| E | soft | dinner | 58 | 08:19 |
| E | soft | lunch | 49 | 09:19 |
| E | hard | breakfast | 23 | 05:34 |
| E | hard | dinner | 97 | 17:59 |
| E | hard | lunch | 62 | 13:37 |
| E | soft | dessert | 23 | 05:49 |
| E | soft | dinner | 90 | 16:20 |
| E | soft | lunch | 93 | 14:11 |
| E | hard | breakfast | 40 | 06:24 |
| E | hard | lunch | 87 | 14:29 |
| F | soft | dinner | 50 | 14:42 |
| F | soft | lunch | 22 | 18:07 |
| F | soft | lunch | 40 | 15:08 |
| F | hard | lunch | 63 | 19:51 |
| F | hard | dinner | 57 | 19:51 |
| G | soft | dessert | 15 | 08:03 |
| G | soft | dinner | 36 | 14:11 |
| G | soft | lunch | 32 | 17:15 |
| G | hard | breakfast | 30 | 16:17 |
| G | hard | dinner | 66 | 19:55 |
| G | hard | lunch | 55 | 21:02 |
| G | soft | dessert | 14 | 06:28 |
| G | soft | dinner | 42 | 12:52 |
| G | soft | lunch | 31 | 13:04 |
| G | hard | breakfast | 16 | 12:58 |
| G | hard | dinner | 72 | 27:28 |
| G | hard | lunch | 56 | 20:44 |
| H | soft | dessert | 36 | 09:28 |
| H | soft | dinner | 36 | 15:05 |
| H | soft | lunch | 52 | 23:46 |
| H | hard | breakfast | 51 | 19:51 |
| H | hard | dinner | 32 | 19:40 |
| H | hard | lunch | 55 | 25:07 |
| H | soft | dessert | 35 | 08:01 |
| H | soft | dinner | 67 | 12:23 |
| H | soft | lunch | 49 | 18:40 |
| H | hard | dinner | 62 | 26:26 |
| I | soft | dessert | 27 | 11:06 |
| I | soft | dinner | 48 | 12:34 |
| I | soft | lunch | 27 | 14:32 |
| I | hard | breakfast | 32 | 09:01 |
| I | hard | dessert | 11 | 07:19 |
| I | hard | dinner | 56 | 18:02 |
| I | hard | lunch | 39 | 18:18 |
| I | soft | dessert | 20 | 06:24 |
| I | soft | dinner | 32 | 08:08 |
| I | soft | lunch | 37 | 12:35 |
| I | hard | breakfast | 33 | 12:06 |
| I | hard | dinner | 61 | 29:16 |
| I | hard | lunch | 41 | 20:31 |
| L | soft | dessert | 10 | 02:21 |
| L | soft | dinner | 52 | 11:09 |
| L | soft | lunch | 81 | 17:58 |
| L | hard | breakfast | 50 | 24:14 |
| L | hard | dinner | 72 | 16:06 |
| L | hard | lunch | 88 | 26:02 |
| L | soft | dessert | 30 | 04:48 |
| L | soft | lunch | 65 | 14:56 |
| L | hard | breakfast | 62 | 17:37 |
| L | hard | dinner | 71 | 21:24 |
| L | hard | lunch | 70 | 17:11 |
| M | hard | breakfast | 35 | 10:53 |
| M | hard | dessert | 14 | 04:58 |
| M | hard | lunch | 79 | 13:44 |
| M | soft | lunch | 51 | 08:19 |
| M | hard | breakfast | 28 | 10:30 |
| M | hard | dinner | 60 | 24:46 |
| M | hard | lunch | 82 | 18:58 |
| N | soft | dinner | 40 | 08:58 |
| N | soft | lunch | 43 | 09:14 |
| N | hard | breakfast | 18 | 06:35 |
| N | hard | dinner | 40 | 15:05 |
| N | hard | lunch | 52 | 11:45 |
| N | soft | dinner | 44 | 08:32 |
| N | soft | lunch | 54 | 09:04 |
| N | hard | breakfast | 16 | 06:20 |
| N | hard | dinner | 36 | 10:53 |
| N | hard | lunch | 30 | 07:17 |
| O | soft | lunch | 26 | 12:52 |
| O | hard | dinner | 23 | 14:39 |
| O | hard | lunch | 22 | 17:11 |
| O | soft | dinner | 33 | 12:04 |
| O | hard | breakfast | 11 | 07:34 |
| O | hard | dinner | 41 | 11:59 |
| O | hard | lunch | 21 | 13:50 |
| P | soft | dinner | 54 | 10:52 |
| P | soft | lunch | 33 | 09:07 |
| P | hard | breakfast | 58 | 18:12 |
| P | hard | dinner | 55 | 19:03 |
| P | soft | dinner | 58 | 11:12 |
| P | soft | lunch | 39 | 08:54 |
| P | hard | dinner | 50 | 13:41 |
| P | hard | lunch | 50 | 09:24 |
| Q | soft | dinner | 50 | 08:54 |
| Q | soft | lunch | 38 | 12:05 |
| Q | hard | breakfast | 21 | 08:14 |
| Q | hard | dinner | 54 | 15:39 |
| Q | hard | dinner | 42 | 12:45 |
| Q | hard | lunch | 39 | 10:55 |

Table S2 – Videos used to find the threshold per participant, with information on food texture, eating episode, total bites per video, and video duration (in MM:SS format)
