# Supplementary material for: Rule-based systems to automatically count bites from meal videos
Source: Front Nutr. 2024 May 17;11:1343868. doi: 10.3389/fnut.2024.1343868 (PMC11141395; doi:10.3389/fnut.2024.1343868)
Supplement: Supplementary file 3 [file Table_3.docx]

|  | Grid Search | Random Search | Bayesian Optimization | Average Accuracy | Total Duration | Total Bites | Number of Videos |
| --- | --- | --- | --- | --- | --- | --- | --- |
| Grid Search | 1.00 | 0.60 | 0.82 | 0.95 | 0.03 | -0.15 | -0.01 |
| Random Search | 0.60 | 1.00 | 0.41 | 0.75 | 0.15 | 0.10 | 0.13 |
| Bayesian Optimization | 0.82 | 0.41 | 1.00 | 0.88 | 0.03 | -0.01 | 0.21 |
| Average Accuracy | 0.95 | 0.75 | 0.88 | 1.00 | 0.08 | -0.03 | 0.13 |
| Total Duration Seconds | 0.03 | 0.15 | 0.03 | 0.08 | 1.00 | 0.71 | 0.78 |
| Total Bites | -0.15 | 0.10 | -0.01 | -0.03 | 0.71 | 1.00 | 0.79 |
| Number of Videos | -0.01 | 0.13 | 0.21 | 0.13 | 0.78 | 0.79 | 1.00 |

Table S3 – Correlation matrix between the grid search, random search and Bayesian optimization accuracy and, total bites, total video duration, and number of videos per participant
